# Histopathological Changes of Dental Follicles of Impacted Third Molars in Ibb Governorate

**DOI:** 10.7759/cureus.55455

**Published:** 2024-03-03

**Authors:** Mohammed S Al-Dumaini, Al-Kasem M Abbas

**Affiliations:** 1 Department of Oral and Maxillofacial Surgery, Ibb University, Ibb, YEM; 2 Department of Oral and Maxillofacial Surgery, Sana’a University, Sana’a, YEM

**Keywords:** impacted lower third molar, pericoronal pathosis, histopathology, cbct, dental follicle

## Abstract

Objective: There is a lack of data pertaining to the examination of dental follicles (DFs) in asymptomatic impacted lower third molars (ILTMs) using cone beam computed tomography (CBCT) and histopathological analysis in the Yemeni population. The objective of this study was to explore the DFs of asymptomatic ILTMs through radiological (CBCT) and histological analysis.

Materials and methods: This prospective study comprised 60 patients aged 18-50 years with ILTMs. The ILTMs in these patients exhibited a DF with measurements ranging between 3 and 5 mm. CBCT was employed to evaluate the maximum width of the DF surrounding the crown of ILTMs in horizontal, sagittal, and coronal sections. After the extraction, the DFs were examined for any pathological changes and categorized as normal, inflammatory, cystic, or neoplastic. The data analysis was performed using IBM SPSS Statistics for Windows, Version 21 (Released 2012; IBM Corp., Armonk, New York, United States), and the statistical significance was determined by employing chi-square tests with a significance level of 0.05.

Results: In the study, a total of 60 patients were included, with 17 (28.3%) being male and 43 (71.7%) being female. The majority of the patients 26 (43.3%) fell within the age range of 26-35 years. Regarding the angulation of the ILTMs, most of them were mesioangular 45 (75%), followed by vertical 7 (11.7%), horizontal 4 (6.6%), buccoangular 3 (5%), and distoangular 1 (1.6%). Histopathological changes were observed in 44 of the samples (73.3%). The majority of histopathological changes identified in the DFs were dentigerous cysts 26 (59%) followed by odontogenic keratocysts 11 (25%). Thirty-six (81.8%) of histopathological changes were found in females, whereas in only eight samples (18.1%), histological changes were observed in males. This difference was statistically significant (*p*=0.004). However, there were no statistically significant differences observed in the occurrence of histopathological changes based on age, angulation, and follicle size (p>0.05).

Conclusion: Follicles in ILTMs varied significantly based on gender, with a higher occurrence in females, and tooth impaction angle, mainly in the mesioangular position. Furthermore, a follicular size of 3-5 mm was associated with a higher incidence of pathological changes. Hence, histopathologic examination is recommended for surgically removed ILTMs irrespective of the follicle size observed in radiographic images.

## Introduction

An impacted tooth is characterized by its inability to fully or partially emerge in the oral cavity due to an obstruction along its path of eruption. This obstruction may arise from factors such as neighboring teeth, dense bone, fibrous tissue, or the presence of a cyst or tumor. It is worth noting that impacted lower third molars (ILTMs) are commonly observed to be impacted within the oral cavity [1].

The timing of eruption of the third molars can be influenced by multiple factors, including dietary habits resulting in tooth wear, reduction in the crown size in the mesiodistal direction, decreased usage of the chewing muscles, and genetic inheritance. Moreover, existing research suggests a higher incidence of ILTMs in females compared to males [2].

The dental follicle (DF) is an ectomesenchymal tissue that envelops the developing tooth. In some cases involving impacted third molars, the DF has the potential to give rise to cysts such as dentigerous cysts, odontogenic keratocysts, and ameloblastomas [3]. However, relying solely on the radiographic size of the follicle may not accurately ascertain the presence of pathology. Therefore, it is recommended to perform a histological examination of the follicle to evaluate any associated pathology, as the absence of radiographic abnormalities does not ensure the absence of pathology [4].

The decision regarding whether to retain or extract asymptomatic ILTMs is a subject of ongoing scholarly debate. In many cases, third molars are often considered problematic and functionally unnecessary, leading to their frequent extraction [5]. Stephens et al. observed that using a pericoronal radiolucency greater than 2.5 mm as a diagnostic criterion for follicular cysts may result in false-positive findings and an increased prevalence rate [6].

Several studies have examined the follicles of the ILTMs using two-dimensional radiographic images and have found that follicles measuring larger than 2.5 mm may indicate future pathological changes [3,7-12]. However, there is limited research that examines the volume of the DFs using CBCT. In Yemen, there is a lack of data regarding the examination of DFs in asymptomatic ILTMs using CBCT and histopathological analysis. Therefore, the objective of the present study was to examine both the radiographic (using CBCT) and pathological changes associated with DFs of ILTMs with size measurements ranging from 3 to 5 mm. In addition, it aimed to determine the prevalence of histopathological changes in the DFs of ILTMs in relation to the gender and age of the patient, the size of the DF, and the angulation of the ILTMs according to Winter's classification [13].

## Materials and methods

Study design and location

This prospective clinical study was conducted at the Oral Surgery Clinic, Faculty of Dentistry, Ibb University, Ibb Governorate, Yemen, from 2021 to 2022.

Selection criteria

The inclusion criteria used in this study were: patients aged between 18 and 50 years, with fully ILTMs positioned vertically, mesioangularly, horizontally, distoangularly, buccoangularly/linguoangularly, or in an inverted position. The presence of pericoronal spaces was confirmed by panoramic images. The depth of the pericoronal space was assessed using CBCT and classified as ranging from 3 to 5 mm. In addition, patients with a history of systemic diseases were excluded. The participation of the patients who were included in the study depended on their provision of informed consent.

Ethical approval and sample size

The research protocol received approval from the Medical Ethics Committee of the Faculty of Dentistry, Sana’a University (no.15092020). The total sample size was calculated according to Haidry et al. [8] and Barroso et al. [14].

Radiographical assessment and measurement of the follicle size (FS)

**Figure 1:**
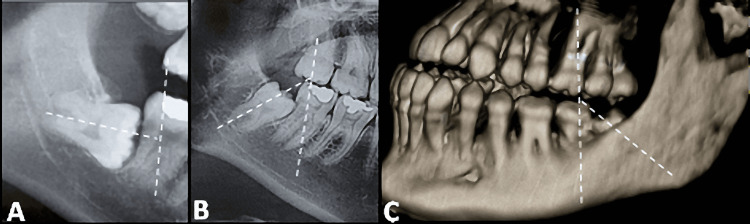
CBCT images illustrate the angulation of the ILTMs (horizontal) as observed in reformatted panoramic view (A) and mesioangular as observed in panoramic view (B) and 3D reconstruction view (C). ILTMs: Impacted lower third molars

CBCT scans of the jaws were obtained using a CBCT machine (PaX-i3D Smart - 3D Imaging System, Korea). The images were then analyzed using BlueSky Software (BlueSky-Plan 4; BlueSky Bio). All ILTMs were classified according to Winter's classification [13]. On the CBCT coronal images of ILTM, two lines were drawn: one along the long axis of the ILTM and another along the length of the second molar. The angle between these two lines was then calculated (Figure 1).

**Figure 2:**
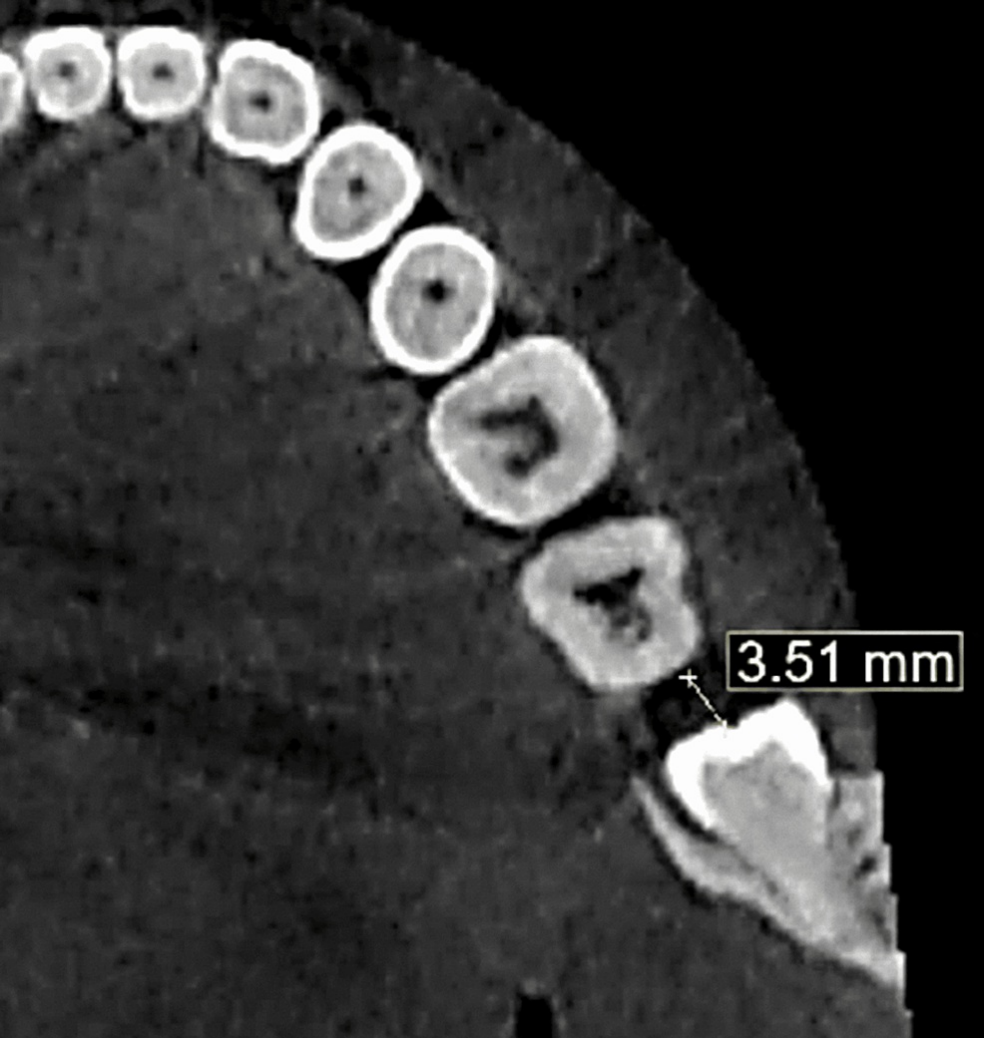
Follicle size measurement as observed in the axial view.

In the "planes" section of the BlueSky software, the sagittal plane was adjusted in the coronal and axial planes to align with the major axis of the ILTM. The slice thickness was increased to 10 mm using the angle measurement tool. Multiple sagittal slices were obtained by reorienting the long axis of the ILTM in the coronal and axial planes. The FSs were measured using the distance tool of the BlueSky software. This measurement was performed for all the selected sagittal sections, and the results were expressed in millimeters (Figure 2). Patients with an FS of 3-5 mm were included in the study. These measurements were taken prior to the surgical extraction and histopathological evaluation of the follicle.

To ensure consistency and reliability, the primary investigator underwent an intra-examiner calibration procedure. This involved measuring five pairs of CBCT images twice, with a one-week interval between measurements.

Surgical procedure

Following the administration of infiltration anesthesia and an inferior alveolar nerve block, a triangular-shaped flap was created by making an incision using a scalpel equipped with a number 15 blade. A periosteal elevator was then utilized to raise a full mucoperiosteal flap, allowing for improved access to the impacted tooth. Next, a straight handpiece with appropriate speed and torque was employed to remove bone from the occlusal aspect of the tooth, with copious irrigation of normal saline. After the extraction of the impacted tooth, a thorough debridement of the surgical site was performed to cleanse it. To ensure a smooth surface, a round bur and bone file were used to eliminate any sharp edges from the remaining bone.

The follicle was enucleated using a hemostat and periapical curette and then cleaned with normal saline. The flap was closed using interrupted sutures made of 4/0 Polyglactin, and hemostasis was achieved by applying compression with sterile gauze. The follicular tissue was cleaned with normal saline and sent for histopathology in 10% formalin. The patient was provided with post-surgical instructions in accordance with the clinical guidelines.

Histopathological procedures

The DF specimens underwent processing and sections were obtained from the blocks embedded in paraffin. This was achieved using a rotary microtome and stained using hematoxylin and eosin stains. Subsequently, the slides were assembled into a montage and viewed under a microscope by a histopathologist.

Following slide preparation, the slides were examined under an optical microscope, and microscopic images were captured to observe any cellular alterations. These examinations were carried out by an oral pathologist with over two years of experience in the field. The diagnoses and observations were recorded in a data collection form for each patient.

**Figure 3:**
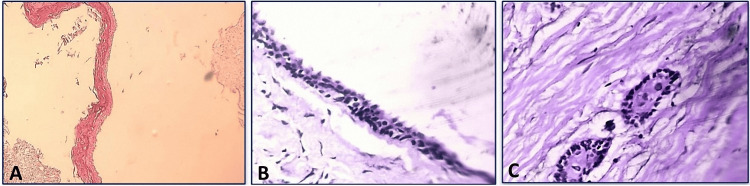
Histopathological analysis images: (A) Stratified squamous epithelium and dense connective tissue, indicating a dentigerous cyst. (B) Uniform epithelial thickness, a hyperchromatic basal cell layer, and wavy surface parakeratin indicating an odontogenic keratocyst. (C) Ameloblastoma with odontogenic epithelium islands within fibrous stroma, wherein the epithelium consists of peripheral palisading cells with hyperchromatic nuclei displaying reverse polarization, along with central stellate reticulum-like cells.

The follicles were classified into two groups: normal follicles, indicating the absence of histopathological changes, and pathological follicles, indicating the presence of histopathological changes, distinguished by distinct characteristics (Figure 3).

## Results

This study involved a sample of 60 patients, 17 (28.3%) were male and 43 (71.7%) were female, within an age range of 18 to 50 years. In terms of size distribution, the samples were categorized as follows: 33 (55%) of the DFs measured between 3 and 4 mm, while 27 (45%) fell within the range of 4.1 to 5 mm. Sixteen of the samples (26.7%) showed no abnormalities, while the remaining 44 (73.3%) exhibited histopathological changes.

Among the 44 histopathologically altered samples (73.3%), the histopathological changes observed in DFs included infected follicles 3 (6.8%), dentigerous cysts 26 (59%), odontogenic keratocysts 11 (25%), and ameloblastoma 4 (9%) (Table 1).

**Table 1:** Distribution of the DFs according to the type of histopathological changes. DFs: Dental follicles

Histopathological changes	Frequency	%
Infected follicles	3	6.8
Dentigerous cysts	26	59
Odontogenic keratocysts	11	25
Ameloblastoma	4	9.01
Total	44	100.0

Among the 44 altered DFs, histopathological changes were identified in 36 (81.8%) samples from females, while only eight samples (18.1%) showed such changes in males. This disparity between the sexes was found to be statistically significant, with a p-value of 0.004 (Table 2).

**Table 2:** Correlation between gender and histopathological changes in DFs. DFs: Dental follicles

Gender	Normal follicles (No.)	Histopathological changes (No.)	Total
Male	9	8	17
Female	7	36	43
Total	16	44	60
Chi-Square	8.374		
p-value	0.004*		

Histopathological changes were found in three age groups: 17 cases (38.6%) in the 18-25 age group, 21 cases (47.7%) in the 26-35 age group, and 6 cases (13.6%) in the 36-50 age group. However, statistical analysis showed no significant difference among these age categories (p-value = 0.423).

Histopathological alterations were observed in various positions of ILTMs based on Winter's classification: 5 (11.3%) vertical, 2 (4.5%) horizontal, 35 (79.5%) mesioangular, and 2 (4.5%) buccoangular. Nevertheless, the statistical analysis did not demonstrate a significant difference among these angulation categories (Table 3). Histopathological changes were observed in two size ranges of the specimens: 23 cases (57.5%) were in the 3-4 mm range, and 21 cases (47.7%) were in the 4.1-5 mm range. However, statistical analysis did not show a significant difference between these size groups (p-value = 0.481).

**Table 3:** Correlation between angulation of ILTMs and histopathological changes in DFs. ILTMs: Impacted lower third molars; DFs: dental follicles

Angulation	Normal follicles (No.)	Histopathological changes (No.)	Total
Vertical	2	5	7
Horizontal	2	2	4
Mesioangular	10	35	45
Distoangular	1	0	1
Buccoangular	1	2	3
Total	16	44	60
Chi-Square	4.399		
p-value	0.355		

## Discussion

The primary aim of this study was to investigate the radiographic and pathological alterations observed in DFs associated with ILTMs, with size measurements ranging from 3 to 5 mm. Additionally, the study sought to ascertain the prevalence of histopathological changes in DFs of ILTMs with regard to gender, age, size of the DF, and the angulation of the ILTMs.

Several studies have investigated the follicles of the ILTMs using two-dimensional radiographic images. These studies have indicated that follicles larger than 2.5 mm may be indicative of future pathological changes [3,7-12]. However, there is a limited amount of research that examines the volume of the DFs using CBCT. Consequently, there are currently no established CBCT reference values for the DF volume of ILTMs. Furthermore, there is a dearth of data regarding the correlation between the DF volume and an increased likelihood of developing or progressing into a cystic or neoplastic condition, which is considered the gold standard for histological analysis. To address these controversies surrounding the management of impacted teeth, the present study aims to analyze both the radiographic (using CBCT) and pathological changes associated with DFs of ILTMs, with measurements ranging from 3 to 5 mm in size.

CBCT has become an indispensable tool in the dental field due to its ability to provide accurate measurements [15,16]. With its three-dimensional imaging capabilities and high-resolution images, CBCT aids in precise diagnosis, treatment planning, and monitoring of dental conditions [17-21]. ‏

The elevated prevalence of pathological alterations among females in our investigation may be attributed to two factors: the relatively smaller size of the female jaw and the greater representation of female patients in our sample. The interplay of these factors may contribute to the observed heightened incidence of pathological changes. Nevertheless, the precise cause of this gender disparity remains undisclosed, as suggested by previous research [3,22].

In this study, it was observed that out of the 60 samples that underwent CBCT scans and histopathological examination, a total of 44 DFs (73.3%) exhibited histopathological alterations. Among this percentage, diverse types of histopathological changes were identified in DFs. These changes included infected follicles 3 (6.8%), dentigerous cysts 26 (59%), odontogenic keratocysts 11 (25%), and ameloblastoma 4 (9%). Our results are comparable with those studies that have shown a higher incidence of pathological changes in ILTMs, such as 50% by Baykul et al. [9], which described cystic changes in 50% of the cases. On the other hand, Haidry et al. [8], and Wali et al. [23] reported pathological changes in 24% and 23.3% of cases respectively, while Güven et al. [2] described a low prevalence rate of 2.3% of cysts and neoplasia and Yadav et al. [11] reported pathological changes in 4.44% of the follicular tissues.

In a retrospective study conducted by Al-Khateeb and Bataineh [24], the aim was to assess the prevalence and specific types of radiographically detectable pathological conditions associated with ILTMs in a Jordanian population. Among the 2,432 ILTMs examined, radiographic evidence of lesions was found in 46.4% of cases. Histological analysis further revealed that all pericoronal radiolucent areas corresponded to either cystic or tumorous formations. The most frequently encountered cyst was identified as the dentigerous cyst, while the most prevalent tumor was the ameloblastoma.

The study revealed that the highest number of patients, accounting for 26 patients (43.3%) of the total, were in the age group of 26-35 years. The second-largest group consisted of 24 patients (40%), who were in the age range of 18-25 years, while the remaining 10 patients (16.7%) were aged 36-50 years. Histopathological changes were more frequently seen in the age group of 26-35 years, with 21 cases (47.7%), while the age group of 18-25 years had 17 cases (38.6%). However, there was no statistically significant difference between these two age groups. These findings suggest that individuals aged 26-35 and 18-25 years are more susceptible to experiencing histopathological changes associated with ILTMs. Reasons for extraction included orthodontic treatment, TMJ pain, and prophylactic purposes. This finding aligns with a study conducted by Vigneswaran and Shilpa [25], who also found that the peak incidence of pathologies occurred in individuals aged 20-30 years.

In this study, we compared the angulation of the ILTMs based on Winter's classification [13]. Our findings demonstrate that ILTMs in a mesioagulated position have a higher frequency of 45 cases (75%) and exhibit a greater tendency for histopathological changes compared to ILTMs in vertical and horizontal positions. Nazir et al. reported that the most common impaction pattern is mesioangular, accounting for 37.6% of cases [26]. Barroso et al. suggested that ILTMs in mesioangular and horizontal positions have a higher propensity to develop larger follicular spaces in specific directions, thereby increasing the likelihood of cyst formation [27]. Similar findings indicate that the dimensions of the DF may be influenced by mandibular morphology and adjacent areas with lower bone resistance.

The limitation of this study was the sample's limited scope, as it was exclusively obtained from a specific location in Ibb Governorate. Consequently, these findings may not be fully representative of the broader Yemeni population. This sampling approach introduces the possibility of selection bias and restricts the applicability of the study's conclusions to other regions or populations within Yemen.

## Conclusions

The size of the follicles in ILTMs exhibited substantial variability in teeth with a histopathological diagnosis, and this variability was primarily associated with gender, with a higher prevalence in females, as well as the angulation of impaction, particularly in the mesioangular position. Additionally, a follicular size ranging from 3 to 5mm was found to be correlated with an increased occurrence of pathological changes. Consequently, DFs obtained from surgically extracted ILTMs should be subjected to histopathologic examination, irrespective of the follicle size observed in radiographic images.

## References

[1] Borle RM (2014). Textbook of Oral and Maxillofacial Surgery.

[2] Güven O, Keskln Keskln, A A, Akal UK (2000). The incidence of cysts and tumors around impacted third molars. Int J Oral Maxillofac Surg.

[3] Rakprasitkul S (2001). Pathologic changes in the pericoronal tissues of unerupted third molars. Quintessence Int.

[4] Eliasson S, Heimdahl A, Nordenram A (1989). Pathological changes related to long-term impaction of third molars: a radiographic study. Int J Oral Maxillofac Surg.

[5] Kaveri GS, Prakash S (2012). Third molars: a threat to periodontal health??. J Maxillofac Oral Surg.

[6] Stephens RG, Kogon SL, Reid JA (1989). The unerupted or impacted third molar--a critical appraisal of its pathologic potential. J Can Dent Assoc.

[7] Chu FC, Li TK, Lui VK, Newsome PR, Chow RL, Cheung LK (2003). Prevalence of impacted teeth and associated pathologies--a radiographic study of the Hong Kong Chinese population. Hong Kong Med J.

[8] Haidry N, Singh M, Mamatha NS, Shivhare P, Girish HC, Ranganatha N, Kashyap S (2018). Histopathological evaluation of dental follicle associated with radiographically normal impacted mandibular third molars. Ann Maxillofac Surg.

[9] Baykul T, Saglam AA, Aydin U, Başak K (2005). Incidence of cystic changes in radiographically normal impacted lower third molar follicles. Oral Surg Oral Med Oral Pathol Oral Radiol Endod.

[10] Saravana GH, Subhashraj K (2008). Cystic changes in dental follicle associated with radiographically normal impacted mandibular third molar. Br J Oral Maxillofac Surg.

[11] Yadav M, Meghana SM, Deshmukh A, Godge P (2011). The wisdom behind third molar extraction: a clinicopathologic study. Int J Oral Maxillofac Pathol.

[12] Kaushal N (2012). Is radiographic appearance a reliable indicator for the absence or presence of pathology in impacted third molars?. Indian J Dent Res.

[13] Winter GB (1926). Impacted Mandibular Third Molars. St Louis: American Medical Book Co. 241-79.

[14] Barroso M, Arriola-Guillén LE, Dutra V, Rodríguez JE, Suárez GR (2023). Evaluation of the follicular space volume of lower third molars with different impaction positions and angulations: a cone-beam computed tomography and histopathological study. Heliyon.

[15] Borzangy S, Alqutaibi AY, Krsoum M, Aljohani R, Qadri O (2023). Evaluation of implant placement risk levels in partially edentulous patients using cone beam computed tomography. Cureus.

[16] Alqutaibi AY, Alassaf MS, Elsayed SA (2022). Morphometric analysis of the midline mandibular lingual canal and mandibular lingual foramina: a cone beam computed tomography (CBCT) evaluation. Int J Environ Res Public Health.

[17] Alqutaibi AY, Algabri RS (2015). Limited evidence suggests high risk of implant failure rates among people with generalized aggressive periodontitis. J Evid Based Dent Pract.

[18] Al-Sarem M, Al-Asali M, Alqutaibi AY, Saeed F (2022). Enhanced tooth region detection using pretrained deep learning models. Int J Environ Res Public Health.

[19] Algabri RS, Altayyar SA, Abo-Alrejal HA, Alsourori AA, Alshaibani DA, Mostafa MH (2023). Effect of primary versus secondary splinting impression techniques on the passive fit of screw-retained implant prosthesis: a randomized clinical trial. Bull Natl Res Cent.

[20] Alqutaibi AY, Aboalrejal A (2017). Zygomatic implants are a reliable treatment option for patients with atrophic maxilla. J Evid Based Dent Pract.

[21] Alqutaibi AY, Esposito M, Algabri R, Alfahad A, Kaddah A, Farouk M, Alsourori A (2017). Single vs two implant-retained overdentures for edentulous mandibles: a systematic review. Eur J Oral Implantol.

[22] Adelsperger J, Campbell JH, Coates DB, Summerlin DJ, Tomich CE (2000). Early soft tissue pathosis associated with impacted third molars without pericoronal radiolucency. Oral Surg Oral Med Oral Pathol Oral Radiol Endod.

[23] Wali GG, Sridhar V, Shyla HN (2012). A study on dentigerous cystic changes with radiographically normal impacted mandibular third molars. J Maxillofac Oral Surg.

[24] Al-Khateeb TH, Bataineh AB (2006). Pathology associated with impacted mandibular third molars in a group of Jordanians. J Oral Maxillofac Surg.

[25] Vigneswaran AT, Shilpa S (2015). The incidence of cysts and tumors associated with impacted third molars. J Pharm Bioallied Sci.

[26] Nazir A, Akhtar MU, Ali S (2014). Assessment of different patterns of impacted mandibular third molars and their associated pathologies. J Adv Med Dent Sci Res.

[27] Barroso M, Arriola-Guillén LE, Rodríguez-Cárdenas YA, Ruíz-Mora GA, Guerrero ME, Flores-Mir C (2018). Tridimensional assessment of the dental follicle dimensions of impacted mandibular third molars using cone-beam CT. J Clin Exp Dent.

